# Developing a Health-Spatial Indicator System for a Healthy City in Small and Midsized Cities

**DOI:** 10.3390/ijerph19063294

**Published:** 2022-03-10

**Authors:** Jiemei Luo, Edwin H. W. Chan, Jinfeng Du, Linxia Feng, Peng Jiang, Ying Xu

**Affiliations:** 1 Department of Building and Real Estate, The Hong Kong Polytechnic University, Hong Kong 999077, China; jiemei.luo@connect.polyu.hk; 2 College of Architecture and Urban Planning, Tongji University, Shanghai 200082, China; 3 Department of Public Administration, Hunan University, Changsha 410082, China; xuyingefface@gmail.com; 4 School of Public Policy and Administration, Xi’an Jiao Tong University, Xi’an 710049, China; jinfeng.du@xjtu.edu.cn (J.D.); uestcfenglinxia@163.com (L.F.); 5 China Center for Urban and Small Town Development, Beijing 100045, China; jiangpeng@ccud.org.cn

**Keywords:** Healthy City, built environment, urban design, small and midsized city, indicator system

## Abstract

A recent examination of the significant role of public health has prompted calls to re-investigate how the urban environment affects public health. A vital part of the solution includes Healthy City initiatives that have been the subject of extensive policies, implications, and practices globally. However, the existing literature mainly focuses on big cities and metropolitan areas, while investigations into small and midsized cities (SMCs) are lacking, and thus reflect the underlying issues of health inequity. This study develops an indicator system for evaluating Healthy City initiatives in SMCs, linking urban design and public health, supported by the analyzed opinions from experts collected using both questionnaires and interviews. The indicator system includes six primary dimensions and 37 variables: urban form and transportation (UFT); health-friendly service (HFS); environmental quality and governance (EQG); community and facility (CF); green and open space (GOS); and ecological construction and biodiversity (ECB). A fuzzy synthetic evaluation technique was used to assess the relative importance of factors, emphasizing the importance of UFT, HFS, and EQG, with importance indexes of 0.175, 0.174, and 0.174, respectively. This indicator system is helpful for SMCs seeking to construct a Healthy City in the future, and is based on urban design and governance inputs and for enhancing the Healthy City knowledge base of cities of varied scales.

## 1. Introduction

The concept of the Healthy City with 11 characteristics was first proposed in 1988 [1]; it received extensive attention followed by physical interventions globally. Overcoming health inequity has recently been highlighted as a major goal, emphasizing conditions in daily life such as the distribution of resources and reducing the health gap [2]. While good health and well-being are part of the United Nations Sustainable Development Goals (UN SDGs), there has been a call for global development addressing the causes of inequality [3]. More evidence from low- and middle-income countries on how urban planning contributes to public health is needed, and particular attention to underprivileged, vulnerable, and easily ignored geographic areas and social groups is necessary [4,5,6], especially in the post-COVID-19 landscape.

Healthy urban design is recognized as an essential issue that includes addressing the design of urban places for the community to address health inequity [2,7,8]. Discussion of the association between the built environment, urban design, and public health is hardly new. However, many previous studies have focused on big cities [9,10,11,12,13], general cities without specific attention to city scale, or urban–rural differences [14,15]. Far more limited literature can be found for small and midsized cities (SMCs). This is possibly caused by limited data openness, with neither environmental data nor health data available for smaller cities [16].

Recent research has revealed unique challenges faced by SMCs and called for a customized understanding of the Healthy City concept for various city sizes. The obesity-increasing rate in SMCs is higher than in both big cities and rural areas [17]. Compared with metropolitan areas, economic factors are a major bottleneck to SMCs’ health development, both for urban design and sanitation facilities [18,19]. The development of SMCs is primarily influenced by their place in the regional transportation plan (e.g., highways) [20,21]. Some small village-like cities are facing a shortage of sanitation facilities [22]. Although SMCs are facing competition from big cities, especially in population attractiveness [19,23], a previous study has revealed that the key attractive factors in big cities, such as diversity, density, and entertainment, have a much weaker effect in SMCs [24]. Some SMCs have greater land and water capacity than big cities, but need a clear guide for population and industry development [25]. Increasing willingness to share health-related data is also important for cities of a smaller scale to promote Healthy City achievements, especially in terms of air quality [16,26]. How urban design and governance could better contribute to the creation of a Healthy City in SMCs thus requires careful exploration. Therefore, this study seeks to extend the understanding of how urban design and governance could better contribute to a Healthy City for SMCs, which have received limited attention in previous studies.

The population line of SMCs varies globally, with resident populations less than 75,000 [27], between 50,000 and 250,000 [28], between 75,000 and 350,000 [29], less than one billion [30,31], or from 500,000 to 2.5 million [24]. However, a rigid population line for defining SMCs is unnecessary, but self-identification based on a function and location perspective is acceptable [32]. This study focuses on developing a health-spatial indicator system for SMCs to better achieve Healthy City development through urban design and governance.

## 2. Design of the Study/Method

**Figure 1:**
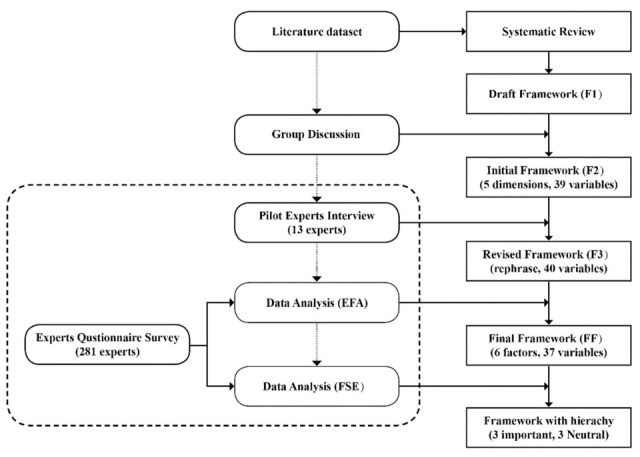
Research Process.

We started with a systematic review of the related literature to identify a proposed factor series for the expert survey. A combined questionnaire and interview were then conducted with relevant professionals to validate the indicator system. Exploratory factor analysis (EFA) and fuzzy synthetic evaluation (FSE) were used to analyze the questionnaire data. The research flow is shown in Figure 1.

Potential academic articles were prepared using Web of Science in February 2021. The full search code for Web of Science is listed as follows:

AB = ((health city OR healthy city) AND (urban OR town OR space OR built environment) AND urban design) AND TS = ((health city OR healthy city) AND (urban OR town OR space OR built environment) AND urban design)

The publishing time was set as 2010 to 2020 (years inclusive). To retrieve an appreciable number of studies, we only selected the papers published in journals with a minimum of three publications on the subject topic. Three academic fields—urban studies, regional urban planning, geography and architecture—were included. The search result included a total of 131 articles from 27 journals. Of these, 23 articles were identified as irrelevant and excluded for the content assessment after the preliminary assessment. After a systematic literature review, a list of criteria related to urban design and Healthy Cities was extracted. After 3 rounds of discussion, 5 significant aspects and 39 factors were chosen. This also comprised the components of the theoretical framework and was further refined for the questionnaire survey. A pilot expert interview was conducted to seek expert comments to validate and refine these criteria as 40 variables to fit the specific topic better. In the pilot study, the experts were invited to remove and propose any variables for the framework. The panel of experts consisted of 13 professionals selected for their expertise in urban design, architecture, urban planning, health services, and social workers. A revised questionnaire was developed with three parts. The first part provided an introduction to the survey purpose, clearly defining the topic range. Second, 40 statements were categorized into five groups, namely health service, UF and function, GOS, environmental quality and energy, and society and governance. The statements were measured on a seven-point Likert scale, with seven indicating the most vital importance. The final part gathered demographic data from the respondents, including socio-economic and career information.

The questionnaire survey was deliberately carried out using a purposive sampling approach to select expert respondents with relevant knowledge and experience on the research topic. The target respondents included scholars who have published on the topic and who have led related research projects. A snowball sampling method was further adopted to enlarge the sample size and gain an exceptionally knowledgeable group. Respondents were requested to send the survey link to their professional networks and colleagues with relevant knowledge or working experience [33,34]. This ensured that the respondents had a certain degree of knowledge of the research topic [35]. The respondents were not limited to academic scholars but included architects, an urban planner, and professionals working in relevant fields who may have had sufficient practical experience to balance professional bias. As the target expert groups were challenging to reach, the snowball sampling method was an efficient approach allowing the possibility of attitude convergence [33,36]. Although this study does not advocate the use of a population line to define SMCs, the level “less than 1 million residents” was provided as a cue for respondents in their consideration. The geographic region was not limited to extensively reflect the research question and provide global experience for further research.

## 3. Theoretical Framework

Urban design contributes to a Healthy City in varied ways. At the beginning of the 21st century, research on health issues and the urban environment mainly focused on reducing non-communicable diseases (NCDs), such as obesity and mental health [21,37,38]. After the COVID-19 pandemic, both NCDs and infectious diseases will require attention in terms of urban environmental interventions [39]. As 54% of the world’s population lives in cities, the role of urban design in promoting a Healthy City needs extensive attention [8]. After a systematic review of the literature on the theory of the Healthy City within the context of urban design, 5 factors with a total of 40 variables were considered for the basic theoretical framework, and they are summarized and described as follows.

### 3.1. Health Services

Health service is a fundamental factor for urban health and typically includes hospitals, healthcare centers, and sanitation services [40,41]. The uneven accessibility of health services for varied socio-economic groups is a key issue for health service provision [4,40,42]. McKee stated that the location of a healthcare center should consider coordinating with real estate acquisition and nearby residential areas [41]. At the community level, the provision of emergency services can offer effective treatment for patients [43], especially for aging people in nursing homes and assisted living, who need intensive service [44].

Disabled and aging people, as marginal and vulnerable social groups, require more consideration and support from healthcare services [45,46]. Typical disabled care design strategies include barrier-free entry for wheelchair users and visually disabled groups, unique handrails, dog-using guides, and altered ground color [46]. Aging, as birth rates have fallen and longevity has increased globally, has become a key urban challenge worldwide. The World Health Organization’s emphasis is on “aligning health systems to the needs of older people” [47]. With this background, emerging age-friendly communities have clear advantages for older residents by catering to their living requirements and mobility, providing more open spaces and accessibility to neighborhood amenities [48,49]. The social attributes of the environment can deeply influence aging people’s health and quality of life [50]. Moreover, the revitalization of old buildings and districts to solve “aging-in-place” should not be ignored [26,51].

### 3.2. Urban Form and Function

The urban structure and transportation infrastructure profoundly influence resident lifestyle and health by encouraging or impeding pedestrianism or cycling [52,53,54,55,56,57]. Layout, density, land-use mix, polycentric forms, and job-housing distance further influence resident lifestyles. The urban transportation infrastructure, including the road networks and connections, and particularly the public transport service, profoundly influence residents’ connectivity and convenience [54,58,59,60,61,62,63,64,65,66]. Residential space, as one vital type of land use, requires the careful consideration of residential density, such as median floors of accommodation and the portion of affordable housing, along with the layout and diversity of community facilities, including ordinary retail facilities and grocery stores [26,48,67,68,69]. Facilities that encourage physical activity, such as sports fields (e.g., swings, basketball courts, handball, and baseball fields), cycling paths, and playgrounds have been revealed to correlate with healthy conditions [69,70,71]. Recreational and entertainment facilities have also been considered as social and cultural facilities, especially those that are public.

### 3.3. Green and Open Space

GOS contributes to urban health in two ways: providing quality pedestrian walking and related physical activities to further contribute to mental health [21]. Typical activities include walking, exercising, and relaxing in parks [72,73]. Urban spaces include parks, open spaces, and sports fields, providing venues for physical activities that reduce obesity [74]. The proximity to and quality of open space also affect healthy urban design and planning [38]. Streetscape, which includes both the formal aspect ratio, sidewalks and the presence of trees, is correlated with walking and related physical behaviors [53,62,75,76]. 

The accessibility and use of GOS contribute to urban health. Detailed indicators include park proximity, distance to nearest GOS, and GOS density [77]. It is necessary to consider the form and distribution of greenspaces and, more specifically, the size of the nearest GOS is associated with walking and usage [11,38]. Distribution is related to socio-economic factors, which needs to be considered for equity and the needs of minorities [78]. The effects of GOS vary based on the different qualities of the greenspace. The current perceptions of crime and disorder strongly affect park quality [79]. Landscape design elements—including lawns, plazas, small lakes, walkways [13], vegetation style [9,68], tree quality [80,81], urban farming [82], roadside vegetation, urban greening features, and environmentally friendly buildings [26,83]—have received extensive attention. Outdoor environment features have also been highlighted from a science-based perspective for urban quality, including sky, tree, and building views [84].

GOSs allow people to experience nature, creating nature relatedness and connectivity [85,86]. This factor can be assessed by the nature and landscape connectivity and the number, size, and density of parks [87]. They provide more opportunities for residents to encounter natural elements, such as trees, animals, and new flowers, further supporting mental health [21]. A “wildlife-inclusive” urban design approach is necessary for coexistence and public health [88]. Maintaining biodiversity thus remains essential for urban health by providing opportunities to encounter wildlife, such as birds [89]. 

### 3.4. Environmental Quality and Energy

Environmental quality further influences physical health through hygiene, sanitation, and energy sustainability. The spatial pattern of PM_10_ can be analyzed and understood to reduce air pollution [90,91]. So, reforming block and wind tunnels can accelerate the dispersion of pollution materials [91,92]. Air pollution, as an essential determinant on human health, could also affected by PM_2.5_, NO_2_, and O_3_ pollutant concentrations [26,93]. Moreover, coastal cities are more sensitive to air pollution-related health and happiness issues than inland cities [94]. Dealing with waste, including litter, undesirable waste, and water pollution, is vital for urban hygiene [18]. Improving toilet facilities and zero-waste systems can contribute to solving related issues [69]. Water sanitation is one vital element related to hygiene and influences the usage of the natural space [67,95]. Storm water facilities, reflected by the length of sewers, surface channels, and areas, together affect water quality [96,97]. Green building elements, such as roof gardens, are one practical approach for improving environmental quality [98].

Sound and thermal aspects are vital environmental factors that have been correlated with living comfort. Serious urban noise nuisances, including screaming, quarrels, and fights, have the potential to negatively impact human health [99]. Thermal comfort and humidity in outdoor places also affect health conditions [12,100]. Both windspeed and UV protection for pedestrians via tree shade could influence urban health [10,101].

### 3.5. Society and Governance

Urban design not only concerns interventions to the physical environment, but also includes governance approaches and public policy. Sense of place, community identity, and social life can be viewed together as a “soft environment” that affects quality of life and human well-being. At the city level, the governance ability and approach deeply affect resident health [102]. At the neighborhood level, accumulating social capital and creating both the sense of and an actual robust community, is vital for local health [53,103]. Spaces increase unplanned social encounters and interaction opportunities contribute to mental health rather than social isolation [104,105]. For historic areas in the city, the conservation of heritage and local culture also needs attention [4]. The role of social interaction in maintaining a healthy lifestyle has been well documented [105]. However, public participation in the decision-making processes related to life and well-being, as well as participation at the community level, tend to be lacking in practice [106,107,108]. As one vital dimension of spatial perception, safety can be influenced by traffic conditions, fire hazards, and varied urban environments. The diversity and vitality of the urban economy profoundly influence urban socio-economic conditions, further affecting urban health [4,42], especially for low-socioeconomic neighborhoods [109].

## 4. Data Analysis

After obtaining the questionnaire data, we used the EFA and FSE techniques to transform this information into six variables in the form of continuous data series before proceeding to the FSE. The primary purposes of an EFA are to identify the principal directions and reduce sub-dimensions in the dataset with a minimal loss of information [35]. EFA is widely used to identify the dimensionality of subjects related to built environments and urban conditions [110,111]. After constructing the variables, we used the FSE to grasp each factor’s importance pattern and ranking [112]. A total of 322 responses were collected in the survey, and respondents who self-selected as “not familiar with the Healthy City concept” were excluded from the data analysis. Data from a final total of 281 questionnaires were used after careful validation examinations (Table 1).

**Figure 2:**
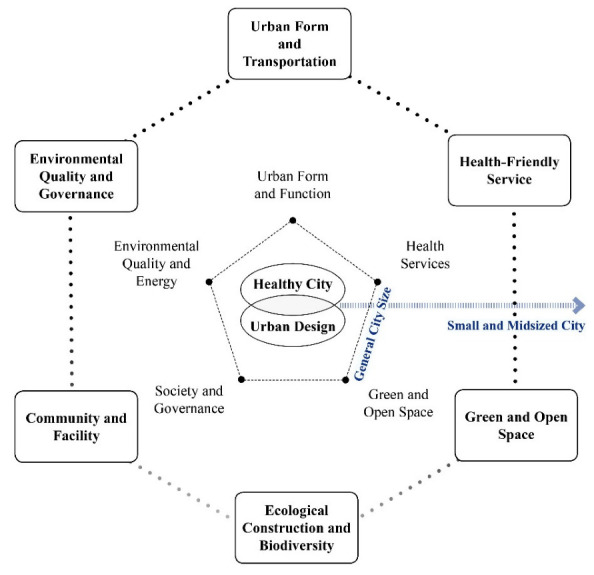
Conceptual framework development.

In the EFA, the Cronbach’s alpha value of 0.967 showed good reliability, indicating a proper consistency among the responses. The Kaiser–Meyer–Olkin (KMO) test (0.952) showed an adequate sampling for the research. SPSS Version 26 was used in the factor analysis. As the questionnaire adopted a seven-point Likert scale, we first examined the mean score of each variable and ranked each of them according to their mean value. No variable had a mean value below 4.0, which represents lower importance in our case; so, all variables were kept for further analysis [113]. After the component rotation, we deleted the variables of education facility, entertainment facility, and cycling facility, because their coefficient value was below 0.5. Six key representative factors, composed of 37 variables, were initially extracted (Figure 2, Table 2).

To further reveal the importance and ranking of each factor, the FSE technique was adopted after categorizing the variables. FSE has been widely used for building composite indicators and establishing assessment frameworks and has a special advantage in generating a ranking index [112]. The FSE technique has the ability to handle complicated evaluations with multi-levels and attributes [114]. Moreover, the method has the potential to objectify subjective opinions from experts [115]. Hence, the FSE was considered very appropriate in this study to ascertain the importance ranking of factors for achieving Healthy Cities in SMCs. FSE was conducted following six key steps [34,112,116,117]:

1. We defined a basic set of variables for each factor based on the EFA results. π = {*f*_1_, *f*_2_, *f*_3_, …, *f_m_*}, where *m* is the number of variables in each factor.
2. We established a set for the grading standard *E* = {*e*_1_, *e*_2_, *e*_3_, …, *e_n_*}. The sets of grading standards were the scale measurements adopted for the study. In this study, the seven-point Likert scale was adopted, where *e*_1_ = extremely low important, *e*_2_ = very low important, *e*_3_ = low important, *e*_4_ = neutral, *e*_5_ = important, *e*_6_ = very important, and *e*_7_ = extremely important.
3. Normalization was applied. We established the weightings for each variable and factor. The weightings (*W*) for each variable and factor were computed from the mean scores [117]: *W**_i_*** = {*w***_1_**, *w***_2_**, …, *w***_m_**}, where (0 ≤ *w***_1_** ≤ 1). Here, the weightings were computed using the following equation [117]:
  (1) $$W_{i}=\frac{M_{i}}{\sum^{\;}M_{ii}}$$
  where *W_i_* is the weighting of a variable or factor; *M_i_* is the mean value of the variable or factor; and ∑*M_ii_* indicates the summation of the mean values of all variables or factors.
4. We computed the membership function (MF) for each variable (second level) and factor (first level), and established a fuzzy evaluation matrix. The matrix was written as *R = (r_ij_) _m×n_*, where *r_ij_* is the degree to which the grading scale *e* satisfies the variable *f_m_*.
5. We computed the weighting vector and the final evaluation matrix:
  (2) $$D=W_{i}\circ R_{i}$$
  where *D* is the final evaluation matrix, and ∘ is a fuzzy composition operator.
6. We normalized the evaluation matrix according to following equation:
  (3) $$Index\;for\;each\;Factor=\overset{7}{\underset{i=1}{\sum }}D\times E$$

From this equation, the index for each factor was determined.

A seven-point Likert scale was used to evaluate the relative importance scores for the variables in this study; so, *E* = {1, 2, 3, 4, 5, 6, 7}. Here, we take Factor 1 “Ecological Construction and Biodiversity” as an example to indicate the analysis process. For the variable “urban farming,” we first calculate its weighting by using Equation (1):

$$W_{Urban\;farming}=\frac{4.51}{\left(4.51+4.78+4.82+\ldots +5.1\right)}=0.133$$

Then, the MF of the variable is determined. The survey results indicated that 2.5% of the respondents asserted that the relative significance of “urban farming” is of extremely low importance, 5.7% of the respondents were also of the opinion that this measurement item is of very low importance, 14.6% of the respondents insisted that this variable is of low importance, while 28.5%, 23.5%, 15.3%, and 10% of the respondents stated their assessment of its relative importance as neutral, important, very important, and extremely important, respectively. As such, the MF for “urban farming” is given by the following equation:

$${\mathrm{MF}}_{Urban\;farming}=\frac{0.025}{ELI\left(1\right)}+\frac{0.057}{VLI\left(2\right)}+\frac{0.146}{LI\left(3\right)}+\frac{0.285}{N\left(4\right)}+\frac{0.235}{LI\left(5\right)}+\frac{0.153}{VI\left(6\right)}+\frac{0.100}{EL\left(7\right)}$$

$$D_{F1}=\left(0.025\;0.057\;0.146\;0.285\;0.235\;0.153\;0.100\right)\circ \left(\begin{array}{l} 0.025\;0.057\;0.146\;0.285\;0.235\;0.153\;0.100\\ 0.025\;0.036\;0.103\;0.228\;0.317\;0.164\;0.128\\ 0.025\;0.028\;0.100\;0.249\;0.285\;0.174\;0.139\\ 0.014\;0.021\;0.117\;0.192\;0.292\;0.203\;0.160\\ 0.021\;0.039\;0.128\;0.181\;0.324\;0.178\;0.128\\ 0.014\;0.036\;0.089\;0.214\;0.285\;0.217\;0.146\\ 0.011\;0.011\;0.085\;0.217\;0.310\;0.171\;0.196 \end{array}\right)=(0.019\;0.032\;0.109\;0.223\;0.293\;0.180\;0.143)$$

and the weightings of the 37 variables and five factors, as well as their MFs, are computed (Appendix A).

The factor index is calculated according to Equation (3), as follows:

(F1)=(0.019 0.032 0.109 0.223 0.293 0.180 0.143)×(1, 2, 3, 4, 5, 6, 7)=4.85

After completing the same calculation for all data, the final results of the indices and importance levels for all factors are shown in Table 3.

## 5. Findings and Discussions

**Figure 3:**
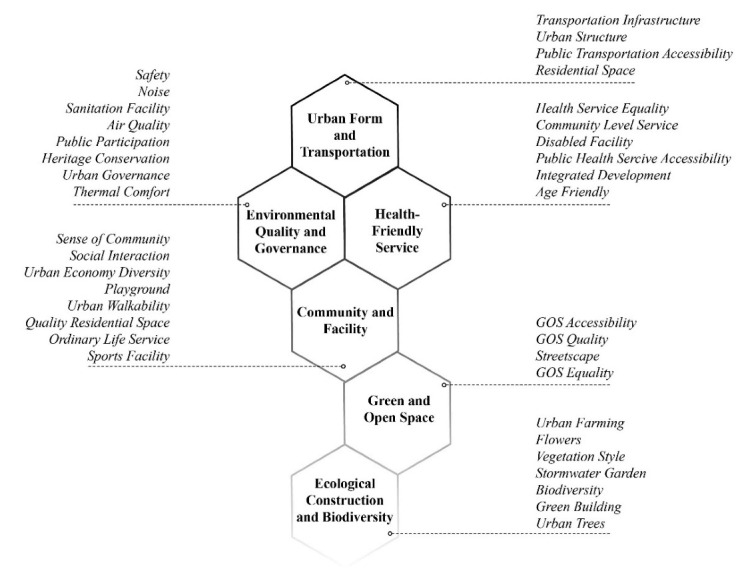
Spatial-health indicator framework for a Healthy City in small and midsized cities.

Table 2 and Figure 3 show the analysis results with components of the indicator systems, including each factor’s composition with the rankings. The importance index is clearly indicated in Table 3. We found six factors with an importance of hierarchy as follows: urban form and transportation (UFT), environmental quality and governance (EQG), and health-friendly service (HFS) were important; community and facility (CF), green and open space (GOS), and ecological construction and biodiversity (ECB) were of neutral importance. The small mean value indicates relative importance but does not mean that the factor would not contribute to a Healthy City. We ranked the variables according to their factor loadings within each factor, as this indicates to what extent it can explain that factor [34,35]. Detailed explanations are discussed below.

### 5.1. Urban Form and Transportation

Factor 6 has the highest index (5.73)—that is, urban form and transportation was revealed as the most critical factor among the six factors (Table 3). Factor 6 accounts for 7.382% of the total variance in the factor analysis. Urban form and transportation refers to factors affecting the overall urban structure, including the transportation system, land use, and structure and layout; that is, it sets the city’s fundamental structure and further affects various aspects of human behavior and urban life, thus having an impact on urban health conditions. Once a city has an advantageous overall UFT, this tends to affect diverse functions that contribute positively to urban health. Two essential components of urban life were highlighted in this factor: transportation and residence. Four variables comprised this factor (Table 2). Transportation infrastructure was revealed as the most important variable, with a factor loading of 0.734. The mean value for this variable was 5.88 (important). Although the mean value of 5.51 was not high, urban structure was ranked second, with a factor loading of 0.692. Although accessibility of public transportation had a factor loading of 0.552 (the third highest), it had the highest mean value of 5.9 and was thus valued as very important. Residential space had a slightly lower factor loading (0.532) and mean value (5.64) than accessibility of public transportation. This variable indicates land supply, density, diversity of housing forms, and overall housing quality.

Consistent with previous studies, transportation infrastructure and public transportation accessibility were revealed as key variables of factor 1 UFT; the results of this study highlight the strong correlation between transportation planning and urban health, reflecting the possible systemic impact this factor has across other determinants of health [118]. The importance to SMCs is probably related to transport injustice, which is caused by the income gap. The transportation infrastructure and urban structure together as UFT profoundly affect the residents’ daily lifestyle, for example, encouraging walking and cycling, and thereby influence public health. If public transportation services are lacking, some residents, especially blue workers, are forced to travel by car, further reducing physical activities [119]. Residential space emphasizing the diversity of housing forms and affordable housing provision for disadvantaged social groups is also necessary.

### 5.2. Environmental Quality and Governance

Factor 2, environmental quality and governance, and Factor 4, health-friendly services, have the same index (5.69), thus ranking as the second and third vital factors that are considered important, respectively (Table 3). Factor 2 accounted for 12.378% of the total variance and was tied for second among the six factors. With an index of 5.69, it consists of eight variables. This factor emphasizes both hard aspects, such as sanitation and pollution, and soft aspects, such as how a city is governed, to enhance urban health in SMCs. The findings suggest a slightly higher importance of environmental quality than governance. Among all of the variables, safety ranked first for both factor loading (0.715) and mean value (6.01). The following three variables were noise, sanitation facility, and air quality, which had factor loadings of 0.666, 0.663, and 0.659, respectively. The mean value for air quality was relatively high, at 5.94. These variables together express the importance of environmental quality. Variables reflecting governance followed the ones related to environmental quality. Public participation (0.577), heritage conservation (0.545), and urban governance (0.544) are the three variables that followed in the ranking. One variable related to environmental quality—thermal comfort—ranked last (0.535), along with a relatively low mean value of 5.41.

The importance of environmental quality possibly reflects the poor development condition of SMCs facing pollution and fundamental sanitation challenges. While most metropolitans are entering the post-industrial and post-modern development period seeking a higher quality of life, many SMCs still struggle to meet essential hygiene provisions. Especially, SMCs tend to have less implementable policies and have minimal data openness regarding air quality [16]. Investment and improvement in air quality through more public engagement and data sharing has the potential to largely enhance urban health in SMCs. Under rapid urbanization, both metropolitan areas and SMCs face challenges in conserving heritage landmarks representing local history and enhancing local character.

### 5.3. Health-Friendly Service

Factor 4, health-friendly services, has the same index (5.69) as Factor 2, environmental quality and governance, indicating a matching importance level. This factor has six variables related to health services of various types and levels, and it accounts for 10.348% of the total variance. Among the six variables, health service equity has the top factor loading of 0.677, emphasizing the importance of health services in different socio-economic areas. Although community-level service and public health service accessibility had lower factor loadings of 0.638 and 0.607, respectively, they had higher mean values of 5.79 and 6.00, respectively. While disabled facilities had a higher factor loading (0.627) than age friendliness (0.546), the mean value for the latter was higher (5.75) than that for the former (5.47).

The importance of health service equity, community service, and public health service accessibility together reflect the observed uneven development level of urban health and the relationship between urbanization level and its ability to provide public services. Community-level service meets daily needs that directly influence quality of life. Concerning vulnerable social groups, the built environment plays a vital role for people with disabilities by providing space for the continuity of daily life. The relatively low ranking of age friendliness probably reflects the more obvious aging phenomenon in metropolitan areas with more significant populations. Even the integrated thinking on health services with overall neighborhood planning showed higher importance than age friendliness. 

### 5.4. Community and Facilities

The final three factors can be considered as being of neutral importance. Factor 3, community and facilities, with an index of 5.43, ranked fourth. Because urban health is related to both health condition and human well-being, it is believed that community and facilities providing social benefits and community service are vital for improving quality of life. This factor component accounted for 11.335% of the total variance and was composed of eight variables. 

Sense of community (0.664) and social interaction (0.662) ranked first and second, respectively, in factor loadings within these variables. A previous study has identified the advantages of SMCs in having closer neighborhood relationships as social capital and connections, particularly for the elderly. Under rapid urbanization and transition, the existing social relationships in SMCs are under threat. Maintaining a neighborly atmosphere for achieving collective identity is vital for SMCs’ healthy development. Urban economic diversity ranked third (0.573), emphasizing the diverse forms and scales of economic enterprises, especially providing more opportunities for individual small businesses. 

Regarding variables that have a direct influence on physical activities, playground had a higher factor loading (0.531) than sport facilities (0.51), while the mean value of sport facilities was higher than playground (5.27 vs. 5.25). Urban walkability and quality residential space had the same factor loadings (0.516), while urban walkability had a higher mean value (5.62 vs. 5.54). Surprisingly, as walkability was the primary domain for achieving urban health that received extensive discussion, it presented relatively lower importance for SMCs. This possibly reflects that walkability is largely determined by urban density and land-use mix [77]. Quality residential space is mainly regarded as open space in the residential estate and regeneration of deteriorating old estates and urban villages. Although ordinary life services did not have a high factor loading (0.512), the mean value (5.79) ranked first among the variables in this factor. Typical ordinary life facilities include retail facilities, grocery stores, and mundane facilities. 

### 5.5. Green and Open Space

Factor 5, green and open space, ranked fifth, with an index of 5.40. GOS is widely recognized as an essential element that contributes to urban health, especially for mental health, in what are known as green health interventions. The neutral importance of GOS possibly reflects the fewer pressures and better mental conditions of SMCs’ living. This factor contains four variables: accessibility, quality, streetscape, and equity. This factor component accounted for 9.44% of the total variance and ranked fifth. 

Among the variables, GOS accessibility had both the highest factor loading (0.684) and mean value (5.52). GOS accessibility, which is closely related to green and nature access, reflects park proximity. Whether the green spaces are close and easily accessible for the public contributes most for SMCs’ public health. GOS quality follows, with a factor loading of 0.652. GOS quality can be reflected by the general perceived safety and comfort, beauty and sky views, or use of detailed landscaping elements. Although streetscape had a higher factor loading (0.623) than GOS equity (0.569), they had the same mean value (5.37).

### 5.6. Ecological Construction and Biodiversity

The final factor (Factor 1), ecological construction and biodiversity, had an index of 4.85. The ecological construction and biodiversity factor had a low index at 4.85 and accounted for 14.612% of the total variance. With seven variables, this factor generally reflects the degree to which people encounter and engage with the natural environment, including urban farming, flowers, vegetation, urban trees, and biodiversity. Storm water gardens and green building are eco-constructions common in urban habitats. 

Among all seven variables, urban farming had the highest factor loading at 0.839. This is possibly because urban farming is a type of urban green space with vital social functions, as urban farms bring different people together, reflecting the social needs of SMCs. For the mean value, only urban trees had a mean value higher than 5, at 5.1, while the rest were below 5, indicating neutral importance. 

The findings of this study contribute to the existing literature in several ways. The proposed indicator system expands the knowledge base of Healthy Cities with the customized consideration of a city’s scale. This study also enhanced our understanding of how built environments affect public health in different development conditions. In addition, both physical and social dimensions of a space need to be considered to better achieve a comprehensive system for urban health from an urban design and governance perspective. 

The importance of hierarchy shows the eager needs of primary fundamental urban functions in SMCs, such as transportation, land structure, and environmental quality. The findings echo the reasons that the WHO put these factors as core indicators instead of expanded ones [67]. In addition, health-friendly service, significantly lower-level, easily accessible healthcare service, presents essential importance. This reflects the issues of SMCs that are usually not in the regional center, not advanced, and short of public service provision. The desire for well-being and happiness has also been highlighted from the importance of social-cultural needs, governance, and community. In contrast, GOSs and ECB were assessed as being of neutral importance. While green spaces, including urban parks, street trees, and experiencing nature have received extensive attention and are well recognized as an essential part of the urban environment that contributes to urban health, comparatively lower importance was revealed in this study on SMCs. It is possible that this is due to SMCs being usually located closer to nature, and because of the residents’ high mobility between urban and rural areas [120,121]; in contrast to providing urban public services, such as public transportation and health services, providing green spaces is not among their major weaknesses. For SMCs, improving overall urban form and transportation infrastructure, providing public services, both in hygiene and health services, are among their priority concerns.

### 5.7. Limitations and Further Study

It is worth mentioning that the framework proposed in this study is not a fixed construct, but rather a flexible option that could be adjusted in local contexts in future longitudinal, empirical, and in-depth studies. The methodology provided in this article could also applied to validate the framework for varied localized contexts with featured variables and hierarchies. While some factors may have a systematic impact across the whole system, more attention could also be paid to the interrelationships among the factors and variables by adopting techniques such as Bayesian network analysis or structural equation modeling. Although we seek to reach and invite experts globally, a possible uneven geographic distribution exists and tends to concentrate in Asia. To avoid this knowledge bias in expert surveys, future empirical studies place more attention on grassroot community perception and the real usage of spaces. SMCs, with different locations, under different development periods, may face varied development conditions and challenges. It is necessary to consider the inner heterogeneity among SMCs for subsequent studies. More in-depth consideration may provide insight into the varied development scenarios that SMCs are facing.

## 6. Conclusions

Small and midsized cities represent the backbone of urban development, and the shortage of relative knowledge on Healthy Cities requires a customized theoretical framework that can better promote the achievement of Healthy Cities. With particular attention on health inequity issues between various city sizes and development conditions, this study developed an indicator framework for constructing a Healthy City in SMCs, with specific consideration of urban design and governance. Comprehensive expert questionnaire surveys and interviews were employed, followed by using EFA to extract key factors and using FSE to further identify the importance of hierarchy among factors. In this way, this article has validated a framework composed of 6 critical factors and 37 criteria.

The indicator system designed in this study provides better understanding of SMCs in achieving urban health and has the potential to strengthen equally the development between metropolitan areas and SMCs. It can also be used as an assessment system for Healthy City performance to identify those urban areas under worse environmental condition that require more health-led improvements and inputs. In addition, the importance hierarchy identified in this study can inform decision-makers about the priorities of planning SMCs.

From a practical perspective, this indicator system may offer long-term impacts by providing valuable insights enabling urban designers and managers to emphasize urban form and transportation, environmental quality, and urban governance in the planning and governing of SMCs to achieve better urban health. For SMCs seeking actions to achieve a Healthy City through urban design, we suggest examining the current city health performance using the framework provided in this study. This study can also inform decision-makers about the importance rankings among diverse variables when undertaking urban development projects, especially for SMCs facing limited resources. The table presented with factor rankings provides supporting materials for planners and managers in decision making to better capture the vital elements in achieving a Healthy City. In the context of dynamic urban changes in SMCs, an adequate understanding of the diverse factors that influence achieving a Healthy City is required across the world.

## Figures and Tables

**Table 1:** Occupational background of the questionnaire respondents.

	Catalog	Portion
**Profession**	Academia, Researcher	59.10%
	Government Staff	4.60%
	Urban Planner, Architect, Landscape Designer	17.40%
	Social Worker	5.30%
	Other	13.60%
**Working Experience**	0–5 years	41.30%
	6–10 years	19.20%
	11–15 years	14.90%
	16–20 years	8.20%
	Over 20 years	16.40%
**Effectiveness of existing framework**	Not effective	0.00%
	Less effective	2.10%
	Neutral	28.50%
	Effective	60.50%
	Highly effective	8.90%

**Table 2:** Mean values, factor loading, percentage of variance explained, and cumulative percentage of the variance of all variables.

Factors	Mean Value	Factor Loading	% of Variance Explained	Cumulative % of Variance
Factor 1 Ecological Construction and Biodiversity			14.612	14.612
26. Urban Farming	4.51	0.839		
24. Flowers	4.78	0.774		
23. Vegetation Style	4.82	0.765		
31. Storm water Garden	4.98	0.714		
27. Biodiversity	4.79	0.712		
34. Green Building	4.95	0.636		
25. Urban Tree	5.1	0.598		
Factor 2 Environmental Quality and Governance			12.378	26.99
39. Safety	6.01	0.715		
32. Noise	5.62	0.666		
29. Sanitation Facility	5.92	0.663		
28. Air Quality	5.94	0.659		
37. Public Participation	5.27	0.577		
40. Heritage Conservation	5.66	0.545		
35. Urban Governance	5.59	0.544		
33. Thermal Comfort	5.41	0.535		
Factor 3 Community and Facility			11.335	38.325
12. Sense of Community	5.29	0.664		
36. Social Interaction	5.34	0.662		
38. Urban Economy Diversity	5.25	0.573		
17. Playground	5.25	0.531		
18. Urban Walkability	5.62	0.516		
11. Quality Residential Space	5.54	0.516		
13. Ordinary Life Service	5.79	0.512		
16. Sports Facility	5.27	0.510		
Factor 4 Health-Friendly Service			10.348	48.673
2. Health Service Equality	5.56	0.677		
4. Community-Level Service	5.79	0.638		
5. Disabled Facility	5.47	0.627		
1. Public Health Service Accessibility	6	0.607		
3. Integrated Development	5.51	0.601		
6. Age Friendly	5.75	0.546		
Factor 5 Green Open Spaces			9.44	58.113
20. GOS Accessibility	5.52	0.684		
22. GOS Quality	5.32	0.652		
19. Streetscape	5.37	0.623		
21. GOS Equality	5.37	0.569		
Factor 6 Urban Form and Transportation			7.382	65.495
8. Transportation Infrastructure	5.88	0.734		
7. Urban Structure	5.51	0.692		
9. Public Transport Accessibility	5.9	0.552		
10. Residential Space	5.64	0.532		
KMO TEST	0.952			
CRONBACH’S ALPHA	0.967			

**Table 3:** Indices and importance levels for all factors.

No.	Factor Groupings	Index	Normalized Index	Importance Level	Ranking
Factor 6	Urban Form and Transportation	5.73	0.175	Important	1
Factor 4	Health-friendly Service	5.69	0.174	Important	2
Factor 2	Environmental Quality and Governance	5.69	0.174	Important	3
Factor 3	Community and Facilities	5.43	0.166	Neutral	4
Factor 5	Green and Open Space	5.40	0.165	Neutral	5
Factor 1	Ecological Construction and Biodiversity	4.85	0.148	Neutral	6

## Data Availability

Not applicable.

## Appendix A

**Table A1:** Weightings and membership functions of all variables and factors.

No.	Variables and Factors	Weightings for Variables/Factors	Membership Functions of Level 1 (Variables)	Membership Function of Level 1 (Factors)
Factor 1	Ecological Construction and Biodiversity	0.169		(0.019, 0.032, 0.109, 0.223, 0.293, 0.180, 0.143)
	26. Urban Farming	0.133	(0.025, 0.057, 0.146, 0.285, 0.235, 0.153, 0.100)	
	24. Flowers	0.141	(0.025, 0.036, 0.103, 0.228, 0.317, 0.164, 0.128)	
	23. Vegetation Style	0.142	(0.025, 0.028, 0.100, 0.249, 0.285, 0.174, 0.139)	
	31. Storm water Garden	0.147	(0.014, 0.021, 0.117, 0.192, 0.292, 0.203, 0.160)	
	27. Biodiversity	0.141	(0.021, 0.039, 0.128, 0.181, 0.324, 0.178, 0.128)	
	34. Green Building	0.146	(0.014, 0.036, 0.089, 0.214, 0.285, 0.217, 0.146)	
	25. Urban Trees	0.150	(0.011, 0.011, 0.085, 0.217, 0.310, 0.171, 0.196)	
Factor 2	Environmental Quality and Governance	0.226		(0.004, 0.017, 0.044, 0.108, 0.232, 0.239, 0.356)
	39. Safety	0.132	(0.004, 0.007, 0.025, 0.075, 0.192, 0.221, 0.477)	
	32. Noise	0.124	(0.007, 0.021, 0.032, 0.110, 0.256, 0.263, 0.310)	
	29. Sanitation Facilities	0.130	(0.000, 0.011, 0.043, 0.089, 0.192, 0.206, 0.459)	
	28. Air Quality	0.131	(0.000, 0.011, 0.039, 0.100, 0.160, 0.231, 0.459)	
	37. Public Participation	0.116	(0.014, 0.028, 0.082, 0.117, 0.288, 0.246, 0.224)	
	40. Heritage Conservation	0.125	(0.000, 0.025, 0.039, 0.114, 0.246, 0.224, 0.352)	
	35. Urban Governance	0.123	(0.007, 0.011, 0.039, 0.132, 0.267, 0.231, 0.313)	
	33. Thermally Comfortable	0.119	(0.004, 0.021, 0.057, 0.135, 0.267, 0.299, 0.217)	
Factor 3	Community and Facilities	0.215		(0.003, 0.016, 0.059, 0.157, 0.264, 0.249, 0.254)
	12. Sense of Community	0.122	(0.000, 0.027, 0.050, 0.187, 0.305, 0.206, 0.225)	
	36. Social Interaction	0.123	(0.004, 0.021, 0.046, 0.178, 0.267, 0.281, 0.203)	
	38. Urban Economic Diversity	0.121	(0.004, 0.021, 0.046, 0.178, 0.267, 0.281, 0.203)	
	17. Playground	0.121	(0.007, 0.007, 0.082, 0.189, 0.281, 0.217, 0.217)	
	11. Quality Residential Space	0.128	(0.000, 0.011, 0.050, 0.146, 0.270, 0.231, 0.292)	
	18. Urban Walkability	0.130	(0.000, 0.011, 0.060, 0.107, 0.253, 0.260, 0.310)	
	13. Ordinary Life Service	0.134	(0.000, 0.004, 0.039, 0.117, 0.189, 0.310, 0.342)	
	16. Sports Facilities	0.122	(0.004, 0.021, 0.075, 0.164, 0.285, 0.246, 0.206)	
Factor 4	Health-friendly Service	0.169		(0.006, 0.009, 0.048, 0.114, 0.246, 0.206, 0.371)
	2. Health Service Equality	0.163	(0.004, 0.021, 0.075, 0.164, 0.285, 0.246, 0.206)	
	4. Community-Level Service	0.170	(0.007, 0.004, 0.060, 0.103, 0.199, 0.199, 0.427)	
	5. Disabled Facilities	0.161	(0.011, 0.021, 0.064, 0.110, 0.270, 0.231, 0.292)	
	1. Public Health Service Accessibility	0.176	(0.000, 0.000, 0.025, 0.082, 0.249, 0.153, 0.491)	
	3. Integrated Development	0.162	(0.007, 0.014, 0.039, 0.164, 0.242, 0.242, 0.292)	
	6. Age Friendly	0.169	(0.007, 0.004, 0.032, 0.103, 0.260, 0.231, 0.363)	
Factor 5	Green and Open Space	0.107		(0.005, 0.010, 0.058, 0.157, 0.275, 0.270, 0.225)
	20. GOS Accessibility	0.256	(0.000, 0.011, 0.043, 0.135, 0.274, 0.302, 0.235)	
	22. GOS Quality	0.247	(0.018, 0.011, 0.064, 0.153, 0.274, 0.253, 0.228)	
	19. Streetscape	0.249	(0.000, 0.000, 0.075, 0.164, 0.292, 0.260, 0.210)	
	21. GOS Equality	0.249	(0.004, 0.018, 0.050, 0.178, 0.260, 0.263, 0.228)	
Factor 6	Urban Form and Transportation	0.114		(0.004, 0.006, 0.034, 0.113, 0.237, 0.257, 0.348)
	8. Transportation Infrastructure	0.256	(0.004, 0.014, 0.025, 0.096, 0.192, 0.253, 0.416)	
	7. Urban Structure	0.240	(0.004, 0.004, 0.057, 0.178, 0.224, 0.238, 0.295)	
	9. Public Transport Accessibility	0.257	(0.000, 0.004, 0.025, 0.075, 0.253, 0.253, 0.391)	
	10. Residential Space	0.246	(0.007, 0.004, 0.032, 0.107, 0.281, 0.285, 0.285)	
